# Curation of a list of chemicals in biosolids from EPA National Sewage Sludge Surveys & Biennial Review Reports

**DOI:** 10.1038/s41597-022-01267-9

**Published:** 2022-04-19

**Authors:** Tess Richman, Elyssa Arnold, Antony J. Williams

**Affiliations:** 1 Oak Ridge Institute for Science and Education (ORISE) Research Participant hosted by EPA. Office of Water, U.S. Environmental Protection Agency, 1200 Pennsylvania Avenue, N.W., Washington, DC 20460 USA; 2 Office of Water, U.S. Environmental Protection Agency, 1200 Pennsylvania Avenue, N.W., Washington, DC 20460 USA; 3 Center for Computational Toxicology and Exposure, Office of Research and Development, U.S. Environmental Protection Agency, 109 T.W. Alexander Dr., Research Triangle Park, NC 27711 USA; 4 Present Address: Office of Pest Management Policy, U.S. Department of Agriculture, 1400 Independence Ave., S.W., Washington, DC 20250 USA

**Keywords:** Environmental monitoring, Environmental chemistry

## Abstract

Section 405(d) of the Clean Water Act requires the US Environmental Protection Agency to review sewage sludge regulations every two years to identify any additional pollutants that may occur in biosolids and to set regulations for pollutants identified in biosolids if sufficient scientific evidence shows they may harm human health or the environment. To date, EPA has conducted eight biennial reviews to identify chemical and microbial pollutants and three national sewage sludge surveys to identify pollutants and obtain concentration data for chemicals found in biosolids. Prior to 2021, there was inconsistent reporting of chemicals identified and EPA did not cumulatively track chemicals in biosolids. Through the efforts presented here, EPA produced a list of 726 chemicals and structure-based classes found in biosolids based on biennial reviews and national sewage sludge surveys. Summary statistics of concentration data are also reported for the 484 chemicals found in the three national sewage sludge surveys. The creation of the Biosolids List supports EPA in assessing the potential risk of chemical pollutants found in biosolids.

## Abstract (summary)

**Table:** 

Measurement(s)	biosolids • chemicals
Technology Type(s)	digital curation
Sample Characteristic - Location	United States of America

## Background & Summary

This paper documents the curation of United States Environmental Protection Agency (EPA) list of chemical pollutants found in biosolids. Biosolids, or treated sewage sludge, are produced from wastewater treatment processes and can be beneficially used or disposed of. Pollutants found in biosolids are reported in biennial review reports [https://www.epa.gov/biosolids/biennial-reviews-sewage-sludge-standards] and in national sewage sludge surveys [https://www.epa.gov/biosolids/sewage-sludge-surveys].

Section 405(d) of the Clean Water Act (CWA) [https://www.epa.gov/biosolids/biosolids-laws-and-regulations] requires the EPA to review sewage sludge regulations every two years to identify any additional pollutants that may occur in biosolids and to set regulations for pollutants identified in biosolids if sufficient scientific evidence shows that they may harm human health or the environment. EPA’s biennial review process is intended to assist in fulfilling this CWA requirement. During the biennial review process EPA collects and reviews publicly available information for (1) pollutants in biosolids that were newly identified during the literature search timeframe; and (2) pollutants in biosolids that were previously identified in EPA national sewage sludge surveys and/or in previous biennial reviews. Information is collected on the occurrence, fate and transport of these pollutants in the environment and their effects on human health and ecological receptors. The types of information collected are needed to conduct risk assessments. EPA has published eight biennial reports (BRs) each covering a two-year timeframe: BR No.1 (2004–2005)^1^, 2) BR No.2 (2006–2007)^2^, BR No.3 (2008–2009)^3^, BR No.4 (2010–2011)^4^, BR No.5 (2012–2013)^5^, BR No.6 (2014–2015)^6^, BR No.7 (2016–2017)^7^ and BR No.8 (2018–2019)^8^.

Additionally, EPA has conducted three national sewage sludge surveys (NSSSs) to date to determine what contaminants may be present in sewage sludge and their concentrations: (1) a National Sewage Sludge Survey in 1988 to gather information on sewage sludge use or disposal practices, and to obtain information on the concentration of over 400 pollutants in the nation’s sewage sludge^9,10^; (2) a NSSS in 2001 (published in 2007) to obtain updated national estimates of dioxins and dioxin-like compounds^11^; and (3) a Targeted National Sewage Sludge Survey (TNSSS) in 2006 (published in 2009)^12,13^ to obtain updated occurrence data on nine analytes identified by EPA in response to the National Research Council (NRC) 2002 report^14^ plus additional contaminants of emerging concern. A second report was published in 2021 on occurrence data for 84 additional contaminants in the TNSSS^15^.

As discussed, EPA’s biennial review process and national sewage surveys are intended to assist in fulfilling the CWA requirement to identify any additional pollutants that may occur in biosolids. The CWA also requires EPA to assess the potential risk of these pollutants and set regulations if sufficient scientific evidence shows that they may harm human health or the environment. Assessing the potential human health and ecological risks associated with pollutants found in biosolids is the top priority for EPA’s Biosolids Program (https://www.epa.gov/biosolids). In consideration of previous efforts to meet the Agency’s statutory responsibility in the CWA Section 405(d) and the 2002 scientific evaluation from the NRC, EPA’s Biosolids Program in the Agency’s Office of Water requested that EPA’s Science Advisory Board (SAB) review its biosolids risk assessment approach which consists of a three-step process to: (1) prioritize the risk assessment of chemical pollutants found in biosolids using computational toxicology; (2) conduct screening-level risk assessments using a customized deterministic model; and (3) conduct probabilistic risk assessments. That SAB review is scheduled for early 2022.

Based on EPA’s national sewage sludge surveys and biennial reports, EPA has reported hundreds of chemical pollutants found in biosolids. Prior to 2021, each survey and biennial report was a “standalone” effort. The curation of the Biosolids List described here allows EPA to work from a full and accurate dataset, developed using a consistent approach and provides EPA a more complete picture to inform its risk assessment efforts. The Biosolids List is publicly hosted on the EPA CompTox Chemicals Dashboard (from here on the “Dashboard”) and allows EPA to take advantage of the functionality of the Dashboard and its integrated data and make the list readily available to the public. The project has leveraged the data contained within the DSSTox database^16^), as well as the skills and experience developed over the past two decades of curating chemistry data, to assemble the Biosolids List^17^. The Dashboard Biosolids list, available at https://comptox.epa.gov/dashboard/chemical_lists/BIOSOLIDS will be updated when future biennial reports or sewage sludge surveys are published.

To develop the list, EPA applied a rigorous approach to assemble and curate chemical names and reported CAS Registry Numbers (CASRNs), along with chemical structures where appropriate, within the ChemReg chemical registration system that houses the DSSTox data^17^. With an assembled dataset, models available through the Comptox Dashboard can be applied to generate predicted physicochemical properties, as well as retrieve fate and transport data, exposure modeling results, and other relevant data, including *in vivo* and *in vitro* toxicity data available for the chemical on that platform. Such an aggregation also allows for integration of other domain appropriate data streams, such as the concentrations of chemicals measured in biosolids.

The identification of chemicals in biosolids by EPA has been underway since the late 1980s and reported in three EPA national sewage sludge surveys and eight biennial reports. To date, a total of 726 chemical substances have been found in biosolids at least once. These unique chemical substances have been subjectively classified into chemical sets using both functional use and structure-based categorization approaches. This approach has identified the presence of pesticides and drugs (and their associated metabolites), cosmetics, and flame retardants, as well as structure-based classes, including polychlorinated biphenyls (PCBs), polybrominated biphenyl ethers (PBDEs), dioxins and dibenzofurans in biosolids. This assembly of data produces a foundational dataset that can provide the basis of a targeted screening collection for mass spectrometry, as well as the basis of risk assessments that are presently underway at EPA.

## Methods

### Curation of chemicals

To compile a list of all chemical pollutants found in biosolids, chemicals were extracted from the three national sewage sludge surveys and eight biennial reports.

All chemicals reported in biennial reports were considered found in biosolids. For chemicals found through national sewage sludge surveys, EPA determined that a chemical was considered detected if the number of detections was greater than zero or if the mean concentration was greater than zero when non-detects were substituted with zero. The calculation of mean concentration in the 1988 NSSS report used a non-parametric substitution method estimation procedure with non-detects substituted for the minimum reporting limit (MRL) or the method detection limit (MDL)^9^. The result produced a mean concentration greater than zero, even for chemicals with zero detections, because the MRL or MDL were greater than zero. Therefore, in subsequent national sewage sludge surveys, a chemical was only considered detected if the number of detections was greater than zero or if the mean concentration was greater than zero when non-detects were substituted with zero.

Chemical names and CASRNs, when reported, were extracted from the three NSSSs and eight BRs. The chemical name formats were standardized for further processing and reviewed using the rules described below. While this was not essential to the registration and curation process, it facilitated further manual review and crosschecking between reports.

1. Isomers. If the chemical name described an isomeric form (e.g., hexachlorodibenzofuran, 1,2,3,6,7,8- or xylene, m-) then the name was reformatted into a more standard form (e.g., 1,2,3,6,7,8-hexachlorodibenzofuran or m-xylene).
2. Acronyms. If a chemical was only reported with an acronym or representative isomeric code (e.g., PCB 209) then the full chemical name, based on searching in the DSSTox database, was added before the acronym (e.g., Decachlorobiphenyl (PCB-209)).
3. Greek Symbols. If a chemical name included a Greek symbol (e.g., β-Sitosterol) the symbol was replaced with the spelling (e.g., Beta-Sitosterol) to ensure that mapping of the chemical would not be affected by character sets.
4. Abbreviations. If an abbreviation was included in the chemical name (e.g., 4-epitetracycline (ETC)) the abbreviation was removed (e.g., 4-epitetracycline).
5. Perfluorinated compounds & salts. Some perfluorinated compounds and salts reported were named according to their charged anionic form (e.g., perfluorooctanoate) rather than their neutral acid forms. For registration purposes neutral forms were adopted (e.g., perfluorooctanesulfonate was modified to be perfluorooctanesulfonic acid). Similarly, all drugs identified as salt forms (e.g., quinine sulfate) were represented in their neutral forms.
6. Combined Chemicals. Since analytical methods commonly cannot distinguish specific isomers, chemicals may be reported in multi-component forms. If a chemical was reported as a combination (e.g., xylene, o- & p- or PCB-107 + PCB-124), the chemicals were separated into two unique chemicals (e.g., o-xylene and p-xylene or 2,3,3′,4′,5-Pentachlorobiphenyl (PCB-107) and 2,3′,4′,5,5′-Pentachlorobiphenyl (PCB-124). This separation allowed for the list to account for all possible unique chemicals detected in biosolids and, specifically, contributed to a growth in the number of PCBs listed. This is discussed in more detail below.
7. Misspellings or Transcription Errors. If a chemical was misspelled or a transcription error was made in the original survey or report (e.g., 1,2,3,4,7,80Hexabromodibenzo-p-dioxin) the name was corrected (1,2,3,4,7,8-Hexabromodibenzo-p-dioxin). Although these changes required expert judgement and some degree of subjectivity, they were necessary to complete the assembly of the list.

After standardizing chemical name formats, and compiling available CASRNs, all chemicals were compared to consolidate duplicates, e.g., if the same chemical was reported in more than one NSSS or BR using different name formats. CASRNs were treated as the primary identifiers. If there were duplicate CASRNs that were reported with different names, the earliest name reported was kept and a note was made indicating that a different name had been used in another source. For example, CASRN 1222-05-5 was reported in BR No.1 with the name “1,3,4,6,7,8-Hexahydro-4,6,6,7,8,8-hexamethylcyclopenta-g-2-benzopyran”, and the same CASRN 1222-05-5 was reported again in BR No.2 with a different name, “Galaxolide (HHCB)”. The chemical is reported in the Biosolids List as 1,3,4,6,7,8-Hexahydro-4,6,6,7,8,8-hexamethylcyclopenta-g-2-benzopyran, CASRN 1222-05-5 with a note “In BR No.2 as ‘Galaxolide (HHCB)’”. It should be noted that Galaxolide is a trade name for the chemical, whereas the selected name is an IUPAC systematic name. Both are mapped to the relevant chemical in the DSSTox database (https://comptox.epa.gov/dashboard/dsstoxdb/results?search=DTXSID8027373#synonyms) along with other synonyms. The list of CASRNs and associated names were registered into the chemical registration system and the crosschecking and validation capabilities of the application were used to confirm and curate the data.

This expert review and curation produced a list of 733 chemical pollutants that have been found in biosolids BRs and NSSSs at least once. Consultation with the Ecological and Health Processes Branch in EPA’s Office of Water identified seven members of the list (nitrate; nitrite; nitrogen; organic-nitrogen, NH4-N, NO3-N; phosphate; phosphorus; and water extractable phosphorus) as “nutrients”; hence, they were removed from the list of chemical pollutants found in biosolids. Nutrients are limited in land-applied biosolids through agronomic rate requirements in the biosolids regulation 40 CFR Part 503, *Standards for the Use or Disposal of Sewage Sludge*. Additionally, nutrients are considered separately from chemical pollutants for risk assessment because they have unique constraints and behaviors in the environment.

**Fig. 1:**
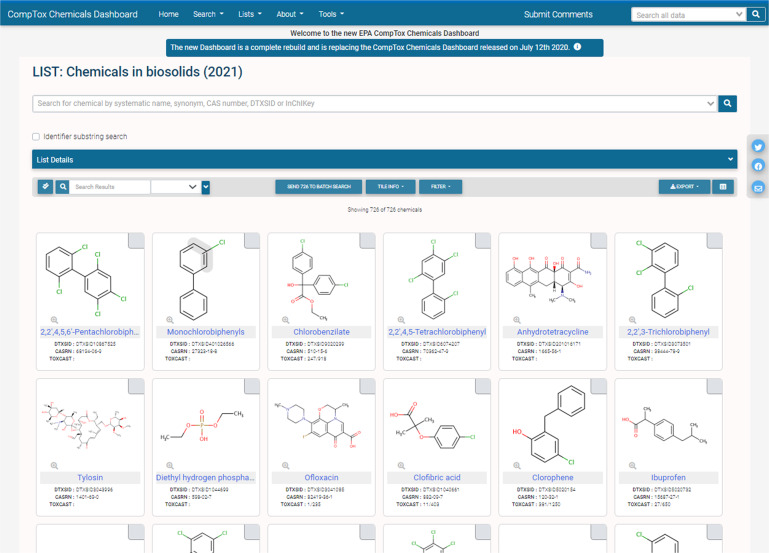
The Biosolids List on the US EPA CompTox Chemicals Dashboard. The list can be downloaded in multiple formats (e.g., Excel, comma-separated values formats). While there are 726 chemicals in the list in total non-structured chemicals are filtered out and not displayed in the list.

The Biosolids List (see Fig. 1) therefore contains 726 chemical pollutants that have been found in at least one instance as reported in a biennial report or national sewage sludge survey.

The Biosolids List reports 191, 223, and 126 chemicals detected in the 1988^9,10^, 2001^11^ and 2006^12,13,15^ NSSS surveys, respectively and 134, 33, 31, 22, 35, 29, 28, and 116 chemicals reported in Biennial Report (BR) No.1^1^, BR No.2^2^, BR No.3^3^, BR No.4^4^, BR No.5^5^, BR No.6^6^, BR No.7^7^ and BR No.8^8^, respectively. Some chemicals were reported in more than one BR or NSSS. A total of 484 chemicals were found in the three NSSSs and 242 chemicals were found in eight biennial reports.

Before the curation process described in this article, an attempt was made to report all chemicals found in biosolids and that list was published in BR. No.7. The list was updated for BR No.8. Appendix B of BR. No.8 reported a total of 511 chemicals in the three national sewage sludge surveys and eight biennial reports. This Biosolids List contains an additional 215 chemicals compared to BR No.8. These are not newly identified chemicals; rather they are chemicals that were reported in an earlier NSSS or BR but were not included in the list published in BR No.8 due to issues with documentation, tracking and formatting. BR. No.8 also included information on when each chemical was “first” or “newly” identified in biosolids based on in which BR or NSSS it was first reported in. After curation of the Biosolids List, changes were made as to when chemicals were considered “newly identified” in biosolids (see Table 1). Changes occurred because chemicals were identified earlier than previously reported. For example, BR No.8 reported 116 “newly identified” chemicals in biosolids. However, after curating the Biosolids List, 41 of the 116 chemicals were previously found in an earlier report and survey. Future biennial reports will report that 75 chemicals were first identified BR No.8.

**Table 1:** Comparison of the number of chemicals reported as ‘newly identified’ in the three national sewage sludge surveys and eight biennial reports in Appendix B of BR. No.8 versus the Biosolids List.

Survey or Report	Number of Chemicals Newly Identified or Reported	
	Appendix B of BR No. 8	Biosolids List
1988 NSSS	92	191
2001 NSSS	12	206
BR No.1 (2004–2005)	94	84
BR No.2 (2006–2007)	24	18
2006 TNSSS	62	52
BR No.3 (2008–2009)	9	2
BR No.4 (2010–2011)	6	8
BR No.5 (2012–2013)	39	35
BR No.6 (2014–2015)	29	28
BR No.7 (2016–2017)	28	27
BR No.8 (2018–2019)	116	75
Total	511	726

Appendix B was published before the effort described in this article. Changes in the number of chemicals considered ‘newly identified’ in the Biosolids List reflect the latest understanding based on the curation process described herein.

EPA has made every effort to be true to what was reported in BRs and NSSSs. In some instances, being true to what was reported has led to double counting. For example, some chemicals have been reported both in families or categories and as unique chemical structures, like PCBs. The PCB homolog Trichlorobiphenyls (DTXSID601026562) is a Markush structure that actually represents 24 unique PCB congeners (PCB 16–39). Biennial reports and sewage sludge surveys have historically included both the homolog and the congeners. Therefore, if the homolog was reported, the Biosolids List contains the homolog and if the individual congener was reported, the Biosolids List contains the congener. The Biosolids List contains the homolog which was reported in BR No.8 and all 24 congeners in that family which were each found in the 2001 NSSS. This could be seen as double counting as the homolog also represents the congeners. The biosolids list contains 726 chemicals counted as unique DTXSIDs, but 11 of those are Markush structures representing families like Trichlorobiphenyls whose members may also be reported individually. EPA will continue to be transparent about how chemicals are reported. When EPA conducts risk screening and assessment, chemicals may be evaluated as individual chemicals or as groups of chemicals.

### Categorization of chemicals

The assembly of the Biosolids List produced a total of 726 chemical substances (see S.I. Table 1). A cursory review of the list indicates that many of these are defined as chemical substances (with distinct structures that would be detectable by mass spectrometry). Some of these substances describe a family of chemicals (e.g., hexachlorobiphenyls which covers a group of 42 unique structures: https://comptox.epa.gov/dashboard/dsstoxdb/results?search=DTXSID001026564#related-substances). Some of the substances are mixtures (e.g., Aroclor 1254, a polychlorinated biphenyl (PCB) mixture containing approximately 21% C12H6Cl4, 48% C12H5Cl5, 23% C12H4Cl6, and 6% C12H3Cl7 with an average chlorine content of 54%)^18^. Data reported in the literature and assembled into the BRs and NSSSs are generally obtained using well defined analytical methods, with PCB chemicals reported as formulae or classes (e.g., octachlorobiphenyls with the formula C12H2Cl8). In order to include all potential PCBs included in a particular class set, the individual classes that would provide various substitutional isomers (e.g., from mono- to nonachlorobiphenyls) were enumerated from Markush representations. The mappings between the individual class parents and their children are provided as supplementary information (see S.I. Table 2).

For the entire list of identified chemicals in biosolids, the potential sources of these chemicals, in terms of exposure and path to being detected in biosolids, was tentatively identified by utilizing a hybrid categorization based on functional use and a structure-based classification approach. An attempt was made to categorize the individual chemicals in terms of their primary membership in a single class, such as pharmaceuticals, cosmetics, pesticides, etc., and clustered appropriately. Many chemicals naturally can be clustered into a primary category (e.g., antibiotics fit most appropriately into the drugs category), whereas other chemicals could fit into multiple categories. Therefore, subjective assignments were made. For example, caffeine is often used in cosmetics, can be used as both a stimulant drug and in pesticides, but likely finds its way into biosolids via human consumption as a food stuff. Similarly, while some of the detected chemicals are listed as a subset of the drugs and drug metabolites category (specifically antibiotics, antimicrobials, and sterols), some of the sterols would be naturally excreted in human waste and some of the antimicrobials would be present in cleaning agents. Although the classification exercise is subjective, the approach is indicative of the most likely use of the chemical substances. The inclusion of structure-based classifications, rather than functional use, segregates the categories of elements (primarily metals), PCBs, dibenzofurans, dioxins, phosphates, per- and polyfluoroalkyl substances (PFAS), PBDEs and polycyclic aromatic hydrocarbons (PAHs). Members of these structure-based classes will also be associated with particular functional uses; for example, members of the PBDE and phosphate classes are used in flame retardants.

The segregation that resulted from the hybrid approach of use and structure classification is included in the supplementary file (S.I. Table 3). The Dashboard has, at the time of writing, a collection of over 300 lists (available at https://comptox.epa.gov/dashboard/chemical_lists/) which are both structure class-based (e.g., polychlorinated biphenyls (https://comptox.epa.gov/dashboard/chemical_lists/PCBCHEMICALS), polycyclic aromatic hydrocarbons (https://comptox.epa.gov/dashboard/chemical_lists/PAHLIST), and elements (https://comptox.epa.gov/dashboard/chemical_lists/elements)) and functional use category-based (e.g., drugs, cosmetics, and flame retardants). In order to attempt a classification of the entire list of chemicals, a Dashboard batch search^19^ was used whereby the DTXSIDs were used as inputs and a number of specific lists were selected in the “Presence in Lists” section of the batch search. The resulting output file included flags indicating membership in particular lists, which was used to assign the chemicals to specific list-category collections. A chemical could be flagged as being present in multiple lists including, for example, caffeine being used as a drug, in cosmetics and in pesticides. Subjective decisions were made to assign chemicals to a specific category but multiple lists were left in the supplementary information file to indicate presence in categories other than that primarily assigned. Those chemicals identified in a list of drugs were segregated into a drug/metabolites category as the Biosolids List contained multiple drug metabolites (e.g., three tetracycline antibiotics were identified: anhydrotetracycline, anhydrochlortetracycline and isochlortetracycline). The drugs/metabolites list was further segregated to include antibiotics and sterols sub-classifications, understanding that some of the sterols are also human hormones and do not necessarily show up in biosolids via a drug source.

An additional flag was incorporated into the export file to indicate “UVCB chemicals” (unknown or variable composition, complex reaction products and biological materials). These were easily identified from the Biosolids List by including InChIKeys in the export^20^, since InChIKeys can only be generated for explicit chemical structures. There are over 30 chemicals identified as being UVCBs. These include Virginiamycin (DTXSID40880080), which is a combination of two specific antibiotic structures; three polymers (polycarbonate, polyethylene glycol and polyethylene terephthalate); and tricresyl phosphate (DTXSID4021391), which is a mixture of three isomeric organophosphate compounds. Of the 32 UVCBs, 13 are PCB classes (e.g., mono-, bi-, trichlorinated biphenyls up to nonachloro-) as well as three PCB mixtures (the Aroclor mixtures). While it is possible that the analytical approach used to detect the PCBs sufficed to quantitate the individual members of a particular Aroclor, in reality it is likely that certain PCBs are detected and simply assigned to be members of the Aroclor mixtures: 1248, 1254 and 1260. Of the chemicals listed, approximately 23% were listed on EPA’s 2016 Chemical Data Reporting (CDR) inventory list (https://www.epa.gov/chemical-data-reporting/access-cdr-data#2020) and an additional flag is included in S.I. Table 3.

### Concentrations of chemicals in biosolids

After the Biosolids List was finalized, any available concentration data for these chemicals were extracted and compiled from the three national sewage sludge surveys (see S.I. Table 4). Concentration data were not extracted from biennial reports because the chemicals were not measured in a nationwide survey conducted by EPA but rather were identified in publicly available literature and may not be representative of chemical concentrations in biosolids nationally.

As discussed previously, when this Biosolids List was assembled, EPA determined that a chemical was considered detected in a NSSS if the mean concentration with non-detects substituted with zero was greater than zero or if number of detections was greater than zero. Chemical concentration data are therefore reported only for chemicals whose mean concentration was greater than zero when non-detects were substituted for zero. Also consistent with the Biosolids List approach, chemical combinations were separated into unique chemicals, for example PCB-107 + PCB-124 was split into the individual components as PCB-107 and PCB 124. The concentration data for each chemical component was reported as the total value of the combination; no attempt was made to apportion the total concentration among components (indicated in notes in S.I. Table 4).

In total, concentration data are reported for 484 chemicals. Concentration data were available for 186, 223 and 126 chemicals and chemical combinations from the 1988, 2001 and 2006 NSSSs, respectively, with 51 chemicals detected and reported in more than one NSSS. For each chemical the parameters ‘Percent Detected’, ‘Mean’, ‘Standard Deviation’, ‘Observed Maximum’ and ‘95^th^ Percentile Concentration Estimate’ are reported, if available. For chemicals detected in the 2006 TNSSS, ‘Minimum’ was also available and reported.

The statistical analysis used to calculate 95^th^ percentile estimates for all NSSSs used the non-parametric substitution method and substituted non-detects for the MRL or 1/2 the MRL except for the 1988 NSSS (discussed in more detail later). The effort described in this article aims to provide what was reported in the sewage sludge surveys. Each survey used a slightly different statistical approach so while concentration data are reported here, further statistical analyses may be necessary when used in risk assessment.

### 1988 National sewage sludge survey concentration estimates

The 1988 NSSS summary statistics were calculated under two different substitution schemes for non-detects: organic chemicals had non-detects substituted for both zero and the MRL; and inorganic chemical non-detects were substituted for zero and the MDL. The concentration percentile estimates reported in this paper and included on the Biosolids List for chemicals detected in the 1988 NSSS are non-detects substituted for the MRL and MDL, depending on the chemical.

Concentration data for chemicals analyzed in the 1988 NSSS are available in two publications. Concentration data for 176 chemicals from the 1988 NSSS were published in the *Technical Support Document for the Round Two Sewage Sludge Pollutants*^9^. The same table of results was published again in Appendix F of the 2006 TNSSS^21^. Both tables were available only as scanned PDFs and values had to be extracted manually rather than exporting the data to a spreadsheet. A visual quality check was done by comparing the published table from both documents. Detailed information regarding how the statistical analysis was conducted for those pollutants analyzed can be found in Appendix B, Statistical Analyses of the National Sewage Sludge Survey Data of the technical support document. Statistical analyses were not conducted for five elemental metals detected less than two times in the 1988 NSSS and concentrations were not reported.

Separate from the 176 chemicals described above concentration data for the ten metals regulated in 1993 were published in the *Statistical Support Documentation for the 40 CFR, Part 503 Final Standards for the Use or Disposal of Sewage Sludge Volume I*^10^. Detailed information regarding how the statistical analyses were conducted for the ten metals can be found in Chapter 7 of the statistical support document.

Of the 191 chemicals detected, concentration data are available for a total of 186 chemicals (176 + 10) from this survey.

### 2001 National sewage sludge survey concentration estimates

The 2001 NSSS summary statistics were calculated three different ways for both organic and inorganic chemicals. In the report EPA substituted non-detects with zero, 1/2 the MRL, and the MRL. The 95^th^ percentile concentration estimates reported in this paper and included on the Biosolids List use non-detects substituted to 1/2 MRL and the MRL for analyzed in the 2001 NSSS. Concentration data reported here were extracted from the *Statistical Support Document for the Development of Round 2 Biosolids Use or Disposal Regulations*^11^. Detailed information on how the statistical analysis was conducted can be found in Section III of the statistical support document.

Of the 223 chemicals detected, concentration data are available for a total of 223 chemicals from this survey.

### 2006 Targeted national sewage sludge survey concentration estimates

The 2006 TNSSS summary statistics were calculated two different ways for both organic and inorganic chemicals. In the reports EPA substituted non-detects for either 1/2 the sample-specific MRL or the “sample-specific ML” which refers to the minimum level adjusted for moisture in each sample when calculating summary statistics e.g., sample-specific MRL. Modeling was used to determine which approach was the best fit for each chemical. The 95^th^ percentile concentration estimates reported for the 2006 NSSS use either the non-parametric or lognormal model.

Statistical analyses for the chemicals analyzed in the 2006 TNSSS were published in two reports. Concentration data for 33 chemicals were published in the *Targeted National Sewage Sludge Survey: Statistical Analysis Report*^13^. Detailed information of the statistical analysis can be found in Appendix C of the statistical analysis report. Data for 82 additional chemicals were published in the *Targeted National Sewage Sludge Survey (TNSSS): Summary Statistics and Estimates of 95*^*th*^ *Percentiles for 84 Additional Analytes*^15^. Detailed information on the statistical analysis for these additional analytes can be found in Section 2 of the report. In-depth statistical analyses were not conducted for eleven pharmaceuticals detected less than two times in the 2006 TNSSS; however, the concentration of the single detection for those chemicals was reported in the *Targeted National Sewage Sludge Survey: Sampling and Analysis Technical Report*^12^.

Concentration data are available for a total of 126 chemicals (33 + 82 + 11 pharmaceuticals) from this survey.

## Data Records

A copy of the data associated with this article are provided as Excel spreadsheets and archived in FigShare^22^. Four files are included:

- Master List of Chemicals Detected in Biosolids: an Excel spreadsheet listing the Biosolids Master List (worksheet 1), all chemical names and CASRNs (worksheet 2) and the list of “nutrients” detected in biosolids.
- Polychlorinated Biphenyl categories and mappings: an Excel spreadsheet containing the categories of (poly)chlorobiphenyls (worksheet 1) and the associated mappings between those categories and the individual category members.
- Categorizations for Chemicals Detected in Biosolids: an Excel spreadsheet containing all chemicals reported in biosolids as well as their classification into multiple categories. Also included in the spreadsheet are the associated CASRNs, DTXSIDs, InChIKeys, and different list memberships based on Dashboard Lists.
- Biosolids Concentration Data from NSSSs: an Excel spreadsheet containing concentration data reported in biennial reviews and sewage sludge surveys (worksheet 1), nutrient concentration data (worksheet 2) and sources of data (worksheet 3).

## Technical Validation

The dataset reported here was rigorously processed and reviewed through the curation procedures that have been reported previously^17^. The extraction of the data from the various biennial reports and sewage surveys provided the seed dataset for review and iterative curation. This included checking identifier mappings and chemical structure representations across multiple public and internal EPA resources.

## Supplementary information

SI Table 2

SI Table 3

SI Table 4

Supplementary Information

SI Table 1

## Data Availability

The data reported in this article are reported in standard Excel format without special code for manipulation.

## References

[1] USEPA, *Biennial Review of 40 CFR Part 503 As Required Under the Clean Water Act Section 405(d)(2)(C) Reporting Period 2005 Biennial Review*, O.o.S.a.T. Office of Water, Washington DC (2006).

[2] USEPA, *Biennial Review of 40 CFR Part 503 As Required Under the Clean Water Act Section 405(d)(2)(C) Reporting Period 2007 Biennial Review*, O.o.S.a.T. Office of Water, Washington, DC (2008).

[3] USEPA, *Biennial Review of 40 CFR Part 503 As Required Under the Clean Water Act Section 405(d)(2)(C) Reporting Period 2009 Biennial Review*, O.o.S.a.T. Office of Water, Washington, DC (2012).

[4] USEPA, *Biennial Review of 40 CFR Part 503 As Required Under the Clean Water Act Section 405(d)(2)(C) Reporting Period 2011 Biennial Review*, O.o.S.a.T. Office of Water, Washington, DC (2015).

[5] USEPA, *Biennial Review of 40 CFR Part 503 As Required Under the Clean Water Act Section 405(d)(2)(C) Reporting Period 2013 Biennial Review*, O.o.S.a.T. Office of Water, Washington, DC (2018).

[6] USEPA, *Biennial Review of 40 CFR Part 503 As Required Under the Clean Water Act Section 405(d)(2)(C) Reporting Period 2015 Biennial Review*, O.o.S.a.T. Office of Water, Washington, DC (2018).

[7] USEPA, *Biennial Review of 40 CFR Part 503 As Required Under the Clean Water Act Section 405(d)(2)(C) Biosolids Biennial Review Reporting Period 2016-2017*, O.o.S.a.T. Office of Water, Washington, DC (2019).

[8] USEPA, *Biennial Review of 40 CFR Part 503 As Required Under the Clean Water Act Section 405(d)(2)(C) Biosolids Biennial Report No 8 (Reporting Period 2018–2019)*, O.o.S.a.T. Office of Water, Washington, DC (2021).

[9] USEPA, *Technical Support Document for the Round Two Sewage Sludge Pollutants*. Washington, DC (1996).

[10] USEPA, *Statistical Support Documentation for the 40 CFR, Part 503 Final Standards for the Use or Disposal of Sewage Sludge Volume I*. Washington, DC (1992).

[11] USEPA, *Statistical Support Document for the Development of Round 2 Biosolids Use or Disposal Regulations*. Washington, DC (2002).

[12] USEPA, *Targeted National Sewage Sludge Survey: Sampling and Analysis Technical Report*. Washington, DC (2009).

[13] USEPA, *Targeted National Sewage Sludge Survey: Statistical Analysis Report*. Washington, DC (2009).

[14] Council, N.R., *Biosolids Applied to Land: Advancing Standards and Practices*. The National Academies Press: Washington, DC (2002).

[15] USEPA, *Targeted National Sewage Sludge Survey (TNSSS): Summary Statistics and Estimates of 95*^*th*^*Percentiles for 84 Additional Analytes*, O.o.S.a.T. Office of Water, Washington, DC (2021).

[16] Williams, A. J. *et al*. *The CompTox Chemistry Dashboard: a community data resource for environmental chemistry*. Journal of Cheminformatics, **9** (2017). 10.1186/s13321-017-0247-6 PMC5705535 29185060

[17] Grulke, C. M. *et al*. *EPA’s DSSTox database: History of development of a curated chemistry resource supporting computational toxicology research*. Computational Toxicology **12** (100096), (2019). 10.1016/j.comtox.2019.100096 PMC7787967 33426407

[18] Force, U.S.A., *The Installation Restoration Program Toxicology Guide*., Aerospace Medical Division, Air Force Systems Command, Wright-Patterson Air Force Base: Ohio. p. 52-1–52-68 (1989).

[19] Lowe, C. M. *et al*. Enabling High-Throughput Searches for Multiple Chemical Data Using the U.S.-EPA CompTox Chemicals Dashboard. *J. Chem. Inf. Model.* **61**(2) (2021). 10.1021/acs.jcim.0c01273 PMC8630643 33481596

[20] Heller, S. R. *et al*. InChI, the IUPAC International Chemical Identifier. *Journal of Cheminformatics* 7 (2015). 10.1186/s13321-015-0068-4 PMC4486400 26136848

[21] USEPA, *Targeted National Sewage Sludge Survey: Statistical Analysis Report, Appendix F: Estimates from the 1988 National Sewage Sludge Survey (NSSS)*, Washington, DC (2009).

[22] Richman T, Arnold E, Williams AJ (2022). figshare.

