# Supplementary material for: Curation of a list of chemicals in biosolids from EPA National Sewage Sludge Surveys & Biennial Review Reports
Source: Sci Data. 2022 Apr 19;9:180. doi: 10.1038/s41597-022-01267-9 (PMC9018786; doi:10.1038/s41597-022-01267-9)
Supplement: Supplementary file 4 — Supplementary Information [file 41597_2022_1267_MOESM4_ESM.docx]

**List of Supplementary Information Files and Descriptions**

***Master List of Chemicals Detected in Biosolids***

An Excel spreadsheet listing the Biosolids Master List (worksheet 1), all chemical names and CASRNs (worksheet 2) and the list of “nutrients” detected in biosolids.

***Polychlorinated Biphenyl categories and mappings***

An Excel spreadsheet containing the categories of (poly)chlorobiphenyls (worksheet 1) and the associated mappings between those categories and the individual category members.

***Categorizations for Chemicals Detected in Biosolids***

An Excel spreadsheet containing all chemicals reported in biosolids as well as their classification into multiple categories. Also included in the spreadsheet are the associated CASRNs, DTXSIDs, InChIKeys, and different list memberships based on Dashboard Lists.

***Biosolids Concentration Data from NSSSs***

An Excel spreadsheet containing concentration data reported in biennial reviews and sewage sludge surveys (worksheet 1), nutrient concentration data (worksheet 2) and sources of data (worksheet 3).
